# Identification of the most indicative and discriminative features from diagnostic instruments for children with autism

**DOI:** 10.1002/jcv2.12023

**Published:** 2021-07-02

**Authors:** Sanna Stroth, Johannes Tauscher, Nicole Wolff, Charlotte Küpper, Luise Poustka, Stefan Roepke, Veit Roessner, Dominik Heider, Inge Kamp‐Becker

**Affiliations:** ^1^ Department of Child and Adolescent Psychiatry, Psychosomatics and Psychotherapy Philipps University of Marburg Marburg Germany; ^2^ Department of Mathematics and Computer Science Philipps University of Marburg Marburg Germany; ^3^ Department of Child and Adolescent Psychiatry Faculty of Medicine TU Dresden Dresden Germany; ^4^ Department of Psychiatry Campus Benjamin Franklin Charité—Universitätsmedizin Berlin Berlin Germany; ^5^ Department of Child and Adolescent Psychiatry and Psychotherapy University Medical Center Göttingen Göttingen Germany

**Keywords:** ADI‐R, ADOS, autism spectrum disorder, diagnostic‐gold‐standard, differential‐diagnosis, machine learning

## Abstract

**Background:**

Diagnosing autism spectrum disorder (ASD) is complex and time‐consuming. The present work systematically examines the importance of items from the Autism Diagnostic Interview‐Revised (ADI‐R) and Autism Diagnostic Observation Schedule (ADOS) in discerning children with and without ASD. Knowledge of the most discriminative features and their underlying concepts may prove valuable for the future training tools that assist clinicians to substantiate or extenuate a suspicion of ASD in nonverbal and minimally verbal children.

**Methods:**

In two samples of nonverbal (*N* = 466) and minimally verbal (*N* = 566) children with ASD (*N* = 509) and other mental disorders or developmental delays (*N* = 523), we applied random forests (RFs) to (i) the combination of ADI‐R and ADOS data versus (ii) ADOS data alone. We compared the predictive performance of reduced feature models against outcomes provided by models containing all features.

**Results:**

For nonverbal children, the RF classifier indicated social orientation to be most powerful in differentiating ASD from non‐ASD cases. In minimally verbal children, we find language/speech peculiarities in combination with facial/nonverbal expressions and reciprocity to be most distinctive.

**Conclusion:**

Based on machine learning strategies, we carve out those symptoms of ASD that prove to be central for the differentiation of ASD cases from those with other developmental or mental disorders (high specificity in minimally verbal children). These core concepts ought to be considered in the future training tools for clinicians.

## INTRODUCTION

Autism spectrum disorder (ASD) is a highly heritable and heterogeneous neurodevelopmental disorder, with characteristic symptoms present in early development and persisting throughout life. The onset of symptoms occurs within the first years of life, leading to impairments of social orientation and reciprocity (Jones & Klin, 2013). Despite early symptoms, ASD is rarely diagnosed before the age of 4 and many children remain undiagnosed until school age or later (Brett et al., 2016; Höfer et al., 2019; Sheldrick et al., 2017). The delay in diagnosis is due to several factors, including a lack of effective screening and a shortage of experienced health care professionals (Ahlers et al., 2019; Carbone et al., 2016). There is a pressing need for tools enabling health care professionals in the primary care sector to identify children for referral to ASD specialists (Abbas et al., 2020). Generation of a valid ASD diagnosis is complex and requires extensive clinical expertise, with behavioral observation as the only basis. The current diagnostic gold‐standard combines an investigator‐based interview for caregivers (Autism Diagnostic Interview‐Revised [ADI‐R]; Rutter et al., 2003) with a clinical behavioral observation (Autism Diagnostic Observation Schedule, ADOS/ADOS‐2; (Lord et al., 2000, 2012). This combination of ADI‐R and ADOS is assumed to enhance diagnostic validity and is recommended for a comprehensive diagnosis of ASD (Kim & Lord, 2012; Risi et al., 2006; Zander et al., 2015). However, administration and evaluation require specific training and are highly time‐consuming. Furthermore, for a (best estimate) clinical diagnosis (BEC), these tools need to be complemented by a careful differential diagnostic examination (Lai et al., 2014), a physical examination, medical history‐taking, and assessment of intellectual abilities (National Collaborating Centre for Women’s and Children’s Health, 2011). Due to an increasing number of individuals requiring a diagnostic examination, waiting lists at specialized institutions continue to expand (“waitlist crisis”) (Kanne & Bishop, 2020), increasingly exceeding specialists' capacities and delaying early diagnoses of ASD. All this has led to a wealth of studies that aimed to develop screening instruments. Due to insufficient operationalization of discriminating items (Brewer et al., 2020), to date there is no evidence for sufficient diagnostic precision (Siu et al., 2016) or functionality and reliability (Thabtah & Peebles, 2019) of screening instruments in the heterogeneous population of autistic individuals. It has become a matter of debate whether and how diagnostic gold‐standards can be reduced to a more cost‐effective, more accessible, and less time‐consuming procedure. In this respect, it is important to identify those behavioral aspects that enable health care professionals to differentiate children with ASD from children with developmental delay, intellectual disability, or other disorders with overlapping symptoms. Knowledge of these most indicative and discriminative behaviors may enable us to develop training tools for clinicians. Such training tools should focus the clinicians' attention on the most relevant aspects of ASD‐related behavior and thus support them to substantiate or rule out a suspicion of ASD leading to an informed decision when to refer an individual to a specialized center.

**Key points**

- The ASD phenotype is heterogeneous and complex, showing symptomatic overlap with other disorders and requiring a specialized diagnostic process conducted by experienced clinicians and a multidisciplinary team.
- An increasing number of individuals demanding a diagnostic examination lead to expanding waiting lists of specialists delaying early diagnosis.
- A subset of diagnostic observations may be sufficient to substantiate a first ADS‐suspicion and thus facilitate clinical decisions whether a child should enter an extensive diagnostic procedure in a specialized institution or be allocated to alternative diagnostic and treatment options.
- Training clinicians in the identification of the most relevant signs of ASD and to realize crucial differences between ASD and non‐ASD may help to optimize early diagnostic decisions.

The present study aimed to carve out subsets of items (“as many as necessary—as few as possible”) that optimally discriminate between groups and asked whether the gold‐standard, combining ADI‐R and ADOS, yields better classification results versus behavioral observation (ADOS) alone. Using machine learning models, we aimed to identify the particular contribution of information directly observed by trained specialists (ADOS) and information provided by parents or caregivers (ADI‐R). Results of this work may lay the foundation for the future training tools that support health care professionals in referring (or not) a given child to a specialized center for ASD.

## METHODS

### Participants

The present project was part of the ASD‐Net, a research consortium funded by the German Federal Ministry of Education and Research (Kamp‐Becker et al., 2017). From this consortium, four specialized centers, where the current diagnostic gold‐standard is applied by specialist clinicians, provided participants' data. All data were collected retrospectively from medical records (retrospective chart review of the period between 2000 and 2019) and analyzed anonymously, with approval from the local ethics committee (Az. 92/20). Due to the retrospective nature of data collection and analysis based on anonymized data, the need for informed consent was waived by the ethics committee. All methods were applied in accordance with relevant institutional and international research guidelines and regulations.

The total sample comprised 1032 cases (mean age = 6.37 ± 3.42) classified as ASD (*N* = 509) or non‐ASD (*N* = 523) based on an International Statistical Classification of Diseases and Related Health Problems 10th Edition (ICD‐10) clinical best estimate diagnosis (BEC). A full description of the sample is provided in Table S1 in the Online Supporting Information. The non‐ASD group comprised a clinically relevant data set with differential diagnosis such as developmental disorders (57%, most frequently developmental disorders of speech and language), attention deficit hyperactivity disorder (ADHD, 15%), or other diagnoses. According to the clinical use of the ADOS modules, that are chosen mainly based on the individual's level of expressive language and chronological age, the total sample consisted of two subsamples including (i) nonverbal children assessed with ADOS module 1 plus the corresponding ADI‐R data and (ii) minimally verbal children assessed with ADOS module 2 plus the corresponding ADI‐R data. Henceforth, these datasets will be referred to as “nonverbal” (module 1) and “minimally verbal” (module 2) cases.

### Subsample 1: Non‐verbal children (ADOS module 1)

The sample of nonverbal children comprised 466 children. Due to young age, ADI‐R and IQ data were only available for a subset of children (ADI‐R: *N* = 198 and IQ‐level estimations according to ICD‐10: *N* = 199).

The ASD group comprised 282 (81% male) children who did not consistently use phrase speech (=nonverbal) and the non‐ASD group included 184 (85% male) nonverbal children. The samples differ slightly according to age, IQ‐level, and ASD‐symptoms, but effect sizes are low (*d* < 0.33, see Table S1 for details). In the ASD group, 61 children had a comorbid disorder. The non‐ASD group included children with a mental disorder (*N* = 102) and children who, after initial suspicion of ASD, did not receive an ICD‐10 axis 1 diagnosis but had mainly a developmental disorder (*N* = 82). Details on the psychopathological sample characteristics are provided in Table S2.

### Subsample 2: Minimally verbal children (ADOS module 2)

The sample of minimally verbal children comprised 566 children. ADI‐R data were available for 304 children. The result of a standardized IQ test (*N* = 246) and clinical estimation of IQ levels (*N* = 370) were available for subsets of children.

The ASD group comprised 227 (83% male) children who used phrase speech but were not verbally fluent (=minimally verbal). The non‐ASD group included 339 (81% male) minimally verbal children without ASD. The ASD group was again slightly older, had lower IQ‐levels and more ASD‐symptoms compared to the non‐ASD group (*d* < 0.34, see Table S1 for details). In the ASD group, 60 children had a comorbid disorder. The non‐ASD group included children with a mental disorder (*N* = 222) and children who, after initial suspicion of ASD, did not receive an axis 1 diagnosis (*N* = 117) (see Table S2 for details).

### Measures

The ADOS (Lord et al., 2000) is an internationally recognized diagnostic instrument that originally consisted of four modules to be administered on the basis of the individual's level of expressive language and chronological age and the appropriateness of assessment materials. There are 29 items in module 1 and 28 items in module 2 that have to be coded. The ADI‐R (Rutter et al., 2003) is a standardized semi‐structured clinical caregiver interview designed to assess ASD‐related symptoms mainly at the age of 4.0–5.0 years. Together these instruments are considered “gold‐standard” assessment measures in the evaluation of ASDs.

### Random forest

To address the abovementioned research questions, we trained random forest (RF) algorithms with (i) the combination of ADOS and ADI‐R data and (ii) ADOS data alone. RFs are ensemble classifiers, based on several decision trees aggregated by majority voting. Each decision tree yields a class prediction considering a random subset of features and a majority vote of all the trees (“the forest”) forms the final classification (Breiman, 2001). For validation purposes, a portion of 25% of the data set was always held out during algorithm training and served as a validation set. Our approach consists of four consecutive steps. (1) Feature selection: a hierarchy of importance for all features was established. (2) Training: stepwise reduced feature models were trained with a 20‐fold cross‐validation using 95% of the data for training and 5% for testing. (3) Evaluation: we then tested the reduced feature models on the hitherto unseen validation data set and determined the “optimal model.” For each model, a weighed ratio of accuracy and complexity (number of variables) was calculated with the choice of the weights favoring simpler models in a 2:1 ratio (i.e., *w*_1_ × AUC + *w*_2_ × complexity where *w*_1_ = 0.35 and *w*_2_ = 0.65). Based on these scores, a final model hierarchy was established and the weighed optimal model was identified. Each model's accuracy (ACC), sensitivity, and specificity are presented as indices of model quality. (4) Comparison: the predictive performance (accuracy) of each reduced feature model was statistically tested against the full features model.

Further details describing the machine learning procedure, including a flowchart (Figure S1) can be found in Appendix S1.

## RESULTS

Model performance indices from the RF models are listed in Table 1. The behaviors associated with the optimal feature subset can be found in Table 2 in descending order of importance. Table S3 in the Online Supporting Information lists items and items abbreviations of ADOS and ADI‐R.

**TABLE 1:** Performance indices of the RF models on the test set (=test) and the previously unseen validation data set (=val) for nonverbal children and minimally verbal children

Number of features	AUC test	ACC test	Sens. test	Spec. test	Youden's J	McN	AUC val	ACC val	Sens. val	Spec. val
Nonverbal children										
ADOS + ADI combination (*N* = 198)										
All 66 features	0.86	0.88	0.79	0.94	0.69	1	0.86	0.81	0.83	0.80
Eighteen features (optimal model) (13 ADOS, 5 ADI‐R)	0.88	0.91	0.83	0.97	0.69	0.92	0.86	0.77	0.74	0.80
Three features (minimal model) (3 ADOS, 0 ADI)	0.79	0.83	0.78	0.81	0.66	0.09	0.76	0.78	0.83	0.73
ADOS alone (*N* = 466)										
All 29 features	0.93	0.92	0.93	0.89	0.54	1	0.88	0.82	0.91	0.72
ADOS six features (optimal model)	0.89	0.90	0.95	0.84	0.50	0.20	0.84	0.78	0.89	0.67
ADOS four features (minimal model)	0.89	0.89	0.93	0.86	0.56	0.09	0.81	0.78	0.93	0.64
Minimally verbal children										
ADOS + ADI combination (*N* = 304)										
All 65 features	0.88	0.89	0.92	0.84	0.51	1	0.90	0.78	0.96	0.62
Sixteen features (optimal model) (11 ADOS, 5 ADI‐R)	0.87	0.89	0.90	0.85	0.52	0.94	0.90	0.80	0.93	0.67
Seven features (minimal model) (5 ADOS, 2 ADI)	0.83	0.85	0.89	0.82	0.51	0.21	0.87	0.74	0.89	0.59
ADOS alone (*N* = 566)										
All 28 features	0.92	0.91	0.96	0.88	0.43	1	0.92	0.85	0.84	0.87
Seven ADOS features (optimal model)	0.91	0.91	0.93	0.89	0.45	0.21	0.89	0.83	0.82	0.84
Six ADOS features (minimal model)	0.91	0.91	0.96	0.86	0.41	0.14	0.90	0.84	0.85	0.82

Abbreviations: ACC, accuracy; ADI‐R, Autism Diagnostic Interview‐Revised; ADOS, Autism Diagnostic Observation Schedule; AUC, area under the curve; J, Youden's Index; McN, McNemar level of significance—each model tested against the full‐feature sets of available features; Sens, sensitivity; Spec, specificity.

**TABLE 2:** Importance ranking from the feature selection process of (a) the optimal number of features for the combined data (ADOS + *ADI‐R*) for nonverbal children (ADOS module 1 and associated ADI‐R data) and minimally verbal children (ADOS module 2 and associated ADI‐R data) (upper row left and right) and (b) the optimal number of features for the behavior observation (ADOS) for nonverbal children (module 1 = M1) and minimally verbal children (module 2 = M2) (lower row left and right)

Nonverbal children	Minimally verbal children
Random forest—ADOS + *ADI‐R*	Random forest—ADOS + *ADI‐R*
1. Use of another's body	1. Stereotyped/idiosyncratic use of words or phrases
2. Pointing	*2. Nodding (ADI)*
3. Gestures	3. Speech abnormalities associated with autism (intonation/volume/rhythm/rate)
4. Unusual eye‐contact	*4. Head Shaking (ADI)*
5. Requesting	5. Shared enjoyment in interaction
6. Response to joint attention	6. Facial expressions directed to others
7. Frequency of spontaneous vocalizations directed to others	7. Amount of reciprocal social communication
8. Integration of gaze and other behaviors during social overtures	8. Spontaneous initiation of joint attention
*9*. *Seeking to share enjoyment with others (ADI)*	9. Overall quality of rapport
*10. Use of other's body to communicate (ADI)*	10. Quality of social overtures
*11. Conventional/instrumental gestures (ADI)*	*11. Circumscribed interests (ADI)*
12. Self‐injurious behavior	12. Descriptive, conventional, instrumental, or informational gestures
13. Functional play with objects	*13. Pointing to express interest (ADI)*
14. Showing	14. Functional play with objects
15. Giving	*15. Conventional/instrumental gestures (ADI)*
*16. Quality of social overtures (ADI)*	16. Imagination/creativity
17. Intonation of voc. or verbalizations	
*18. Hand and finger mannerisms (ADI)*	

Nonverbal children	Minimally verbal children
Random forest—ADOS	Random forest—ADOS
1. Integration of gaze and other behaviors during social overtures	1. Amount of reciprocal social communication
2. Quality of social overtures	2. Shared enjoyment in interaction
3. Spontaneous initiation of joint attention	3. Stereotyped/idiosyncratic use of words or phrases
4. Unusual eye‐contact	4. Facial expressions directed to others
5. Requesting	5. Quality of social response
6. Facial expressions directed to others	6. Quality of social overtures
	7. Functional play with objects

*Note:* Items from the ADI‐R are written in italic letters.

Abbreviations: ADI‐R, Autism Diagnostic Interview‐Revised; ADOS, Autism Diagnostic Observation Schedule.

### Combined ADOS (module 1) and ADI‐R data in nonverbal children

The first step included the identification of the latent feature importance ranking.

**FIGURE 1:**
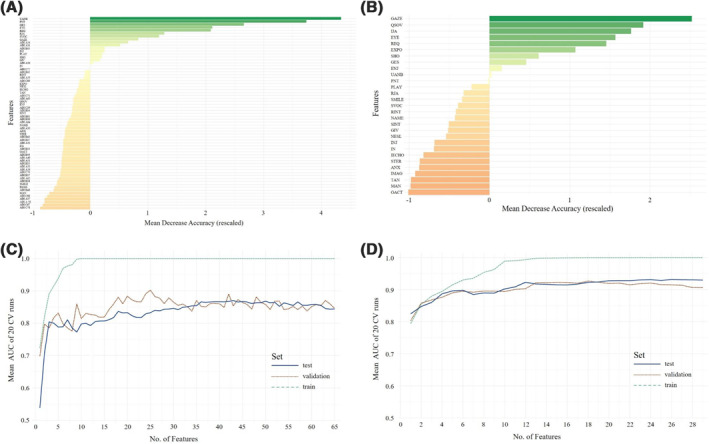
Overall ranking of feature importance in nonverbal children (A) for all 66 items from Autism Diagnostic Observation Schedule (ADOS) module 1 and Autism Diagnostic Interview‐Revised (ADI‐R) combined and (B) for all ADOS module 1 items, referring to the individual mean decrease in accuracy. Mean AUC plotted against the number of model features in nonverbal children (C) for ADOS module 1 and ADI‐R data combined in training, test, and validation sets and (D) for ADOS (module) 1 data alone are depicted. A list of all included items and their abbreviations can be found in Appendix S1

By utilizing the importance hierarchy shown in Figure 1A, RFs for 1 to *n* features were calculated and evaluated on the test data set. The model output from the test set including all 66 variables shows an ACC of 0.88 with 0.79 sensitivity and 0.94 specificity. For independent validation of the classifier, its performance on the validation set was computed and yielded an ACC of 0.81 with 0.83 sensitivity and 0.80 specificity (see also Tables 1 and 2 for an overview).

The ranked feature selection in Figure 1A shows that only few features from the 66 combined ADOS and ADI‐R features contributed strongly to the class prediction, whereas others showed very little predictive value. Plotting the mean AUC of the classifier against the number of features entering the model, a rapid stagnation of model performance in the validation set is seen (see Figure 1C for details).

**FIGURE 2:**
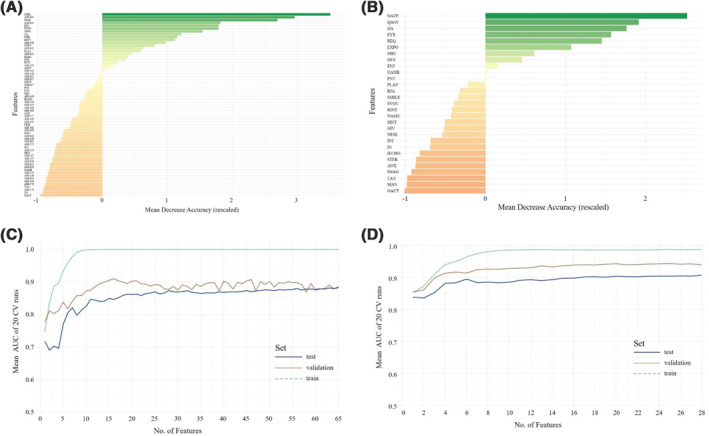
Overall ranking of feature importance in minimally verbal children (A) for all 65 items from Autism Diagnostic Observation Schedule (ADOS) module 2 and Autism Diagnostic Interview‐Revised (ADI‐R) combined and (B) for all ADOS module 2 items, referring to the individual mean decrease in accuracy. Mean AUC plotted against the number of model features in minimally verbal children (C) for ADOS (module 2) and ADI‐R data combined in training, test, and validation sets and (D) for ADOS (module 2) data alone are depicted. A list of all included items and their abbreviations can be found in Appendix S1

The model including 18 features (13 ADOS and 5 ADI‐R items) was considered optimal in the validation set, yielding an ACC of 0.91 with 0.83 sensitivity and 0.97 specificity. This already reduces the feature set, but Figures 1 and 2 indicate additional potential toward a minimal set of features.

As a comparison of the models' performance, McNemar's test for differences in classification error rates showed no advantage of the full‐feature model (66 features) over the weighed optimal model (*χ* ^2^ = 0.008, *p* = .92). The minimal model with three features retained satisfactory performance (AUC = 0.76, ACC = 0.78, sensitivity = 0.83, specificity = 0.73) and showed no statistical difference from the full‐feature‐model (*χ*2 = 2.96, *p* = .09).

### Combined ADOS (module 2) and ADI‐R data in minimally verbal children

First, the latent features importance ranking was evaluated.

According to the feature importance hierarchy shown in Figure 2A, RFs for 1 to *n* features were calculated and evaluated on the test data set. Model output from the test set including all 65 variables showed an ACC of 0.88 with 0.83 sensitivity and 0.90 specificity. Evaluation on the validation set yielded an ACC of 0.83 with 0.74 sensitivity and 0.83 specificity.

The ranked feature selection shown in Figure 2A indicates that only few of the 65 items from the combined ADOS and ADI‐R contributed strongly to the class prediction, whereas others held little predictive value. Plotting the mean AUC of the classifier against the number of features entering the model, we again found a rapid stagnation of model performance in the validation set (see Figure 2C).

The model including 16 features (11 ADOS and 5 ADI‐R items) showed optimal performance in the validation set, yielding an ACC of 0.80 with 0.93 sensitivity 0.67 specificity. As above, Figure 2A and 2C suggests additional potential for feature reduction.

McNemar's test showed no advantage of the full‐feature model (65 features) over the 16‐feature model (*χ*2 = 0.005, *p* = .94). Performance indices for a minimal model containing only seven features (including 5 ADOS and 2 ADI‐R items according to the variable ranking) were also examined on the validation set, yielding an ACC of 0.74 with 0.89 sensitivity and 0.59 specificity and no statistical difference from the full‐feature model (*χ* ^2^ = 1.57, *p* = .21).

### ADOS (module 1) data in nonverbal children

First, a feature hierarchy was established (see Figure 1B) followed by entering 1 to *n* features into separate models.

The model including all 29 ADOS module 1 items showed an ACC of 0.92 with 0.93 sensitivity and 0.89 specificity in the test set. On the validation data set, the performance of the classifier dropped to ACC = 0.82 with 0.91 sensitivity and 0.72 specificity. Figure 1D shows the mean AUC of the classifier against the number of features entering the model. The optimal number of features in nonverbal children was 6. With only six features, the classifier achieved an ACC of 0.90 with 0.95 sensitivity and 0.84 specificity in the test set and an ACC of 0.78 with 0.89 sensitivity and 0.67 specificity in the validation set.

Statistical comparison of the models via McNemar's test showed no advantage of the full‐feature model over the six‐feature model (*χ* ^2^ = 2.60, *p* = .11). Even reduction to four features (ACC = 0.78 with 0.83 sensitivity and 0.73 specificity) did not yield statistical inferiority compared to the full‐feature model (*χ* ^2^ < 3.01, *p* > .08).

### ADOS data (module 2) in minimally verbal children

For module 2, again the subsequent RFs were calculated. Figure 2B shows the feature importance ranks.

The full‐feature model (28 ADOS items) yielded an ACC of 0.91 with 0.96 sensitivity and 0.88 specificity in the test set and an ACC of 0.85 with 0.85 sensitivity and 0.84 specificity in the validation set. Figure 2D shows the mean AUC of the classifier against the number of features entering the model. Reducing the number of features, the model including seven features performed optimally with an ACC of 0.91, 0.93 sensitivity, and 0.89 specificity in the test set and an ACC of 0.83, 0.82 sensitivity, and 0.84 specificity in the validation set.

McNemar's test showed that the seven‐feature model performed equally well as the full‐feature model (*χ* ^2^ < 1.56, *p* > .20). Even another feature could be subtracted, given that a minimal six‐feature model (ACC = 0.84, 0.85 sensitivity, and 0.82 specificity in the validation set) still yielded similar performance as the full‐feature model (*χ* ^2^ = 2.51, *p* = .14).

## DISCUSSION

The present work aimed to identify the most important items from a behavioral observation (ADOS) and a clinical interview (ADI‐R) in a well‐characterized clinical population of children. Using machine learning, we evaluated subsets of diagnostic features from both instruments that were most effective in discriminating groups of nonverbal children and minimally verbal children with ASD from children with other mental disorders or developmental delays. We show that focusing attention on a few diagnostic features may yield sufficiently high quality in the classification decision compared to the full item set contained in ADOS and ADI‐R. Future aim of the present work is to break down these most discriminative subsets of diagnostic items into their underlying mechanisms or processes and translate them into a low‐threshold training tool for clinicians.

With this goal in mind, we identified models with a minimum number of features that did not significantly underperform relative to more elaborate models that included considerably more features and performed optimally in terms of prediction performance related to model complexity (i.e., searching the best accuracy with the least number of features). The statistical equality of the minimal model to the optimal model further corroborates the hypothesis that a reduction of complexity of the diagnostic procedure may be possible. However, the diagnostic instruments—both ADOS and ADI‐R—cannot simply be abbreviated, as, for example, ADOS codes are attained throughout the observation session and are not strictly tied to single subtasks (Lord et al., 2012). This leads to the conclusion that we need to focus on the optimal models that allow for more complexity along with even higher accuracy. Based on these optimal models, by using the underlying concepts of the diagnostic items, we can develop tools for pediatricians and other health care providers training them to realize crucial differences between ASD and non‐ASD. This is not a new idea, as to date there are websites and online tools that attempt to train primary‐care clinicians with reasonable success that promise “earlier detection and lower […] age of referral for evaluation, ultimately allowing families to access early intervention and promote better outcomes for our patients with ASD” (Schrader et al., 2020, p. 307). However, this training is time‐consuming (8‐h course including a video library of more than 24 toddlers) and limited to very young age. Our work adds to the existing literature by further shifting the focus toward efficient and specific training tools for clinicians for different age ranges—away from the more parent‐based (mobile) information tools, such as home videos and so on, that have also been proposed for early screening of ASD (Tariq et al., 2018; Young et al., 2020).

### Combination of ADOS and ADI‐R‐data

For the combined ADOS and ADI‐R data, three features in nonverbal children and seven features in minimally verbal children are sufficient to reach a prediction accuracy that is statistically equal to any model containing more features. However, the optimal classifier required 18 features in nonverbal children and 16 features in minimally verbal children. These models performed optimally in terms of prediction performance versus complexity, that is, best accuracy with least number of features.

In minimally verbal children, the ADI‐R information seems to have less impact on the diagnostic decision than the ADOS. In nonverbal children, however, the ADI‐R seems to contribute to the specificity of a diagnostic decision. Regarding the ADI‐R, it has long been understood that retrospective reports are subject to problems of memory and interpretation, including in studies of ASD (Andrews et al., 2002; Hus et al., 2011). Despite these limitations of retrospective inquiry, parental concerns can index clinically relevant behavioral problems (Chawarska et al., 2007; Glascoe, 2003) and parents may even detect clinically informative behaviors based on their day‐to‐day observations more readily than do clinicians (Sacrey et al., 2018). Parents' concerns about developmental issues should thus be seriously considered, as they do seem to reliably detect the presence of global developmental deficits (Filipek et al., 2000). ASD‐related concerns of parents, however, need to be critically considered during interviewing, as they may lead to an overestimation of ASD‐symptoms and thus a biased report in parents (Havdahl et al., 2017).

For nonverbal children, ADOS items appeared most indicative of ASD according to the RF classifier. Particularly items indicating social orientation (“use of another's body,” “pointing,” and “gestures”) but also information from the reciprocal social interaction domain (“unusual eye contact,” “requesting,” and “response to joint attention”) are most powerful in differentiating ASD from non‐ASD cases. In children with some language, we find language/speech peculiarities (“stereotyped language” and “speech abnormalities associated with autism”) in combination with facial and nonverbal expressions (“nodding,” “head ahaking,” and “facial expressions directed to others”) and reciprocity (“shared enjoyment in interaction”) to be the most important items. Again, ADOS items appear to predominantly drive the differentiation of ASD from non‐ASD cases.

### Only ADOS‐data

For ADOS data alone, similar results were observed: models containing four (nonverbal children) and six (minimally verbal children) features perform similar to the full‐feature model. Relying on the ADOS alone, information from the reciprocal social interaction domain of the ADOS seems essential for class prediction, as almost all items in the optimal models stem from this domain. This may indicate that observations from the social interaction domain have a relatively more pronounced role in classification of ASD and thus more utility in observation‐based diagnosis of ASD.

### Comparison of the combined diagnostic instruments (ADOS and ADI‐R) versus behavior observation (ADOS) only

Classification performance of our reduced feature models is within the range of previous reports of sensitivity and specificity measures of the ADOS and ADI‐R (Randall et al., 2018). In nonverbal children, we find a well‐balanced relation between sensitivity and specificity for models from the combined ADOS and ADI‐R data, whereas the ADOS models yielded higher sensitivity but lower specificity. This observation is in line with previous work showing that the ADOS classifications can have low specificity particularly in children with other mental disorders or developmental issues (Molloy et al., 2011; Zander et al., 2015). This was almost reversed for minimally verbal children, where we find high sensitivity but low specificity for the combination of ADOS and ADI‐R and a well‐balanced relation of both in the ADOS models (optimal and minimal models). Furthermore, in very young children, those with developmental delay or anxiety disorders, parental reports (ADI‐R) perform much worse than clinical behavior observations (ADOS) compared to BEC (Chawarska et al., 2007; Gray et al., 2008; Sacrey et al., 2018). Thus, the trained interviewer/clinician should be well aware of factors that may influence the performance of the ADI‐R cutoff and integrate parent accounts with information from other sources (Havdahl et al., 2017).

From our results, favoring models with higher sensitivity over models with higher specificity, we conclude that for the development of a training tool we need to focus on slightly different behavioral aspects in nonverbal and minimally verbal children. In nonverbal children, particular attention should be payed to the observation of social orientation and reciprocal social interaction. In minimally verbal children, the observation of peculiarities in speech and language, nonverbal communication but also reciprocal social interaction should be trained along with the investigation of parents regarding nonverbal communication (nodding, head shaking, pointing, etc.).

## STRENGTHS AND LIMITATIONS

One major advantage of the present study lies within the clinically relevant data set from a clinical group comprising various psychiatric diagnoses that are difficult to distinguish from ASD. The non‐ASD group consists of a sample of clinic‐referred participants with relevant ASD differential diagnoses, like developmental disorders, ADHD, separation anxiety disorder of childhood, other behavioral and emotional disorders with onset usually occurring in childhood and adolescence or disorders of social functioning with onset specific to childhood and adolescence.

Comparable to most other ASD diagnostic validation studies, one limitation is that the outcome criterion (BEC of ASD vs. non‐ASD) was not independent of the features used for building the prediction algorithms. Although this research design may be criticized, there is little to no alternative as to date there is no independent external criterion replacing BEC. We approached the circularity problem by relying on BEC that included multiple sources of information beyond ADOS and ADI‐R. Aim of the present study was to identify those features (behavioral aspects as assessed by ADOS and ADI‐R) that best predict class membership as opposed by validation studies that aim to test accuracy, sensitivity, and specificity of the diagnostic instrument itself. Given that the feature selection process was central to the present study, the circularity of the diagnostic criteria and the features used in the models is considered acceptable.

Another limitation may be the exclusive use of RF as a method of machine learning while other authors tested diverse methods (e.g., Levy et al., 2018). Although 25% of the data set were held out as an independent validation set, further (international) studies need to evaluate the classifier's ability to generalize to completely new and unseen data to determine its clinical value.

## CONCLUSION

The present work aimed to improve the diagnostic procedure by analyzing the importance of each item from existing diagnostic tools (ADOS and ADI‐R). We suggest to translate these subsets of items into their underlying concepts and then are used these to create an independent training tool that enables clinicians in primary care to distinguish the core, most relevant diagnostic features in children at risk of ASD. This should support evaluation and substantiation of a first ADS‐suspicion and thus facilitate the decision whether a child should enter an extensive diagnostic procedure in a specialized institution or be allocated to alternative diagnostic and treatment options. In order to provide early and valid diagnoses, and considering that neither parent's reports nor observations by non‐specialists are sufficiently sensitive and specific, we need training tools that pediatricians and other health care providers can employ to realize crucial differences between ASD and non‐ASD.

## CONFLICT OF INTEREST

Prof. Dr. Poustka has received payment for consulting or speaking fees from Shire, Takeda, Roche,and InfectoPharm. She receives research funding from the BMBF, DFG, and EU and royalties from Hogrefe, Kohlhammer, and Schattauer. Prof. Dr. Roessner has received payment for consulting and writing activities from Lilly, Novartis, and Shire Pharmaceuticals; lecture honoraria from Lilly, Novartis, Shire Pharmaceuticals, and Medice Pharma; and support for research from Shire Pharmaceuticals and Novartis. He has carried out clinical trials in cooperation with the Novartis, Shire, Servier, and Otsuka companies. The remaining authors declare no potential conflict of interest.

## ETHICS STATEMENT

All data were collected retrospectively from medical records (retrospective chart review of the period between 2000 and 2019) and analyzed anonymously, with approval from the local ethics committee (Az. 92/20). Due to the retrospective nature of data collection and analysis based on anonymized data, the need for informed consent was waived by the ethics committee. [Corrections made on 22 June 2022, after first online publication: This Ethics Statement has been added in this version.]

## Supporting information

Supplementary Material

## Data Availability

The data are not publicly available due to medical confidentiality but are available from the first author on request pending the approval of the coauthors.
