# Supplementary material for: Identification of the most indicative and discriminative features from diagnostic instruments for children with autism
Source: JCPP Adv. 2021 Jul 2;1(2):e12023. doi: 10.1002/jcv2.12023 (PMC10242833; doi:10.1002/jcv2.12023)
Supplement: Supplementary file 1 — Supplementary Material [file JCV2-1-e12023-s001.docx]

**Online Supporting Information for:**

***Identification of the most indicative and discriminative features from diagnostic instruments for Children with Autism***

**Appendix S1.** Methods.

Data preparation: ADOS and ADI-R instruments are indicative of increasing symptom severity via codes of 0, 1, 2 and 3. Additional information about peculiar or abnormal behavior is indicated via codes of 7 or 8 (Lord et al., 2012, p. 17). Following the ADOS and ADI-R manual instructions, codes of 7 and 8 were recoded to 0 and codes of 3 were recoded to 2. All ADOS items were included in analyses but only the 37 ADI-R items were included in the analyses without domain D (Abnormality of Development Evident at or Before 36 Months), as these items do not address symptomatology of ASD.

Machine Learning analyses were based on four steps, which are described in detail below.

**First,** to create a hierarchy of importance for the features (ADOS and ADI-R items), the random forests permutation-based variable importance scores were used, based on 20 random forests consisting of 400 decision trees each. A 20 fold cross-validation was carried out on the training data. By saving every run’s importance hierarchy, each variables’ rank was identified.

**Second,** having access to the robust variable ranking (for n=65 features from combined ADOS and ADI-R data and n=29 features from ADOS data, respectively), a training of reduced feature models including sets of 1 to *n* features ({1},{1,2},{1,2,3}…{1,2,…n}) was carried out. We added single features stepwise into the models according to their rank. Model performance was assessed via the area under the receiver operating characteristic (ROC) curve (AUC). Utilizing the Youden Index, the optimal threshold (where the AUC is at its maximum) was identified. The Youden Index can be used to summarize the performance of a diagnostic test by evaluating its discriminative power (Youden, 1950). During model training, this index was calculated for each threshold of the ROC curve and the point where it achieved a maximum was referred to as the ‘optimal’ threshold. This particular threshold was then used for model validation. To examine the optimal number of variables, the resulting *n* models were compared using both AUC and balanced ACC given the Youden Index as determined in the prior cross-validation process during training. It represents the one point on the ROC curve for which the distance to random guessing (i.e., AUC=.5) is maximal and thus leads to the best classification result that is least likely to happen by chance. The point also represents the class boundary and is thus integrated in the subsequently created models as the threshold for decision making. After computing the AUC and balanced ACC for the *n* models, yet another hierarchy ordering these results was established, based on the idea of information criteria such as Akaike (AIC) and Bayesian information criterion (BIC). These criteria are widely used in statistics to determine the best performing model out of a set of models with different dimensions. Each models performance indices and its number of variables were scaled to the unit interval, then weighted and summed resulting in an individual score for each model (the choice of the weights emphasizes simpler thus less complex models in a 2:1 ratio – i.e. w1*AUC+w2*complexity where w1=0.35 and w2=0.65). Based on these scores, a final model hierarchy was established, and the best performing model, with a still acceptable complexity, was identified.

In a **third** step, we tested the reduced feature models on the hitherto unseen validation set (25% of the full data set) with regards to their classification performance – i.e., separating ASD from non-ASD cases.

The **fourth** and last step was the comparison of the predictive performance of the reduced feature models in terms of the predictive performance of the classifier. We used the McNemar test, a non-parametric statistical test for paired comparisons that can be applied to compare the performance of two machine learning classifiers (Dietterich, 1998), to determine differences in classification error rates between the “optimal model” comprising the optimal number of features against the “full-feature model” for both data bases (combined ADOS and ADI-R data, and ADOS data alone) respectively. This was complemented by the search for a “minimal-feature model” that contained as many variables as needed to exceed the p=0.05 threshold of significant differences compared to the “full-feature model”.

**Figure S1**. Flow diagram of the steps in the machine learning process.


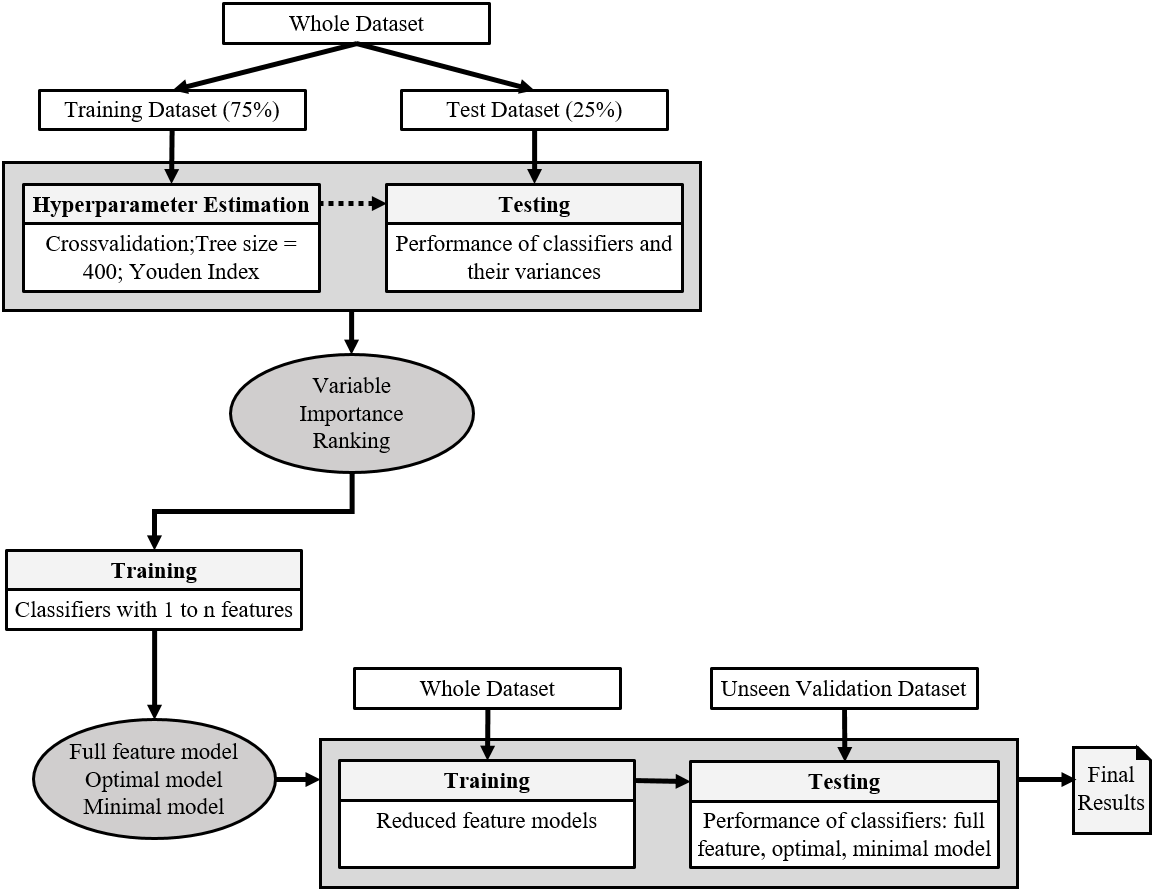


**Table S1**: Sample characteristics for nonverbal (ADOS module 1) and minimally verbal (ADOS module 2) children.

| **Nonverbal children (ADOS Module 1)** | | | | | | | | |
| --- | --- | --- | --- | --- | --- | --- | --- | --- |
|  | ASD | | Non-ASD | | t-Test | | |  |
|  | N | M (SD) | N | M (SD) | t | df | p | ES |
| Age | 282 | 5.8 (4.6) | 184 | 5.1 (3.0) | -1.99 | 463.9 | 0.047 | 0.18 |
| IQ- Level | 118 | 5.3 (0.9) | 81 | 4.9 (1.2) | -2.28 | 136.7 | 0.024 | 0.33 |
| ADOS SA | 273 | 17.1 (6.8) | 184 | 8.0 (14.5) | -9.01 | 455 | 0.001 | 0.86 |
| ADOS RRV | 273 | 5.7 (20.1) | 184 | 2.5 (10.5) | -1.98 | 455 | 0.048 | 0.18 |
|  |  |  |  |  |  |  |  |  |
| ADI-R A | 138 | 17,7 (6.2) | 46 | 14.2 (6.5) | -3.30 | 182 | 0.001 | 0.55 |
| ADI-R B | 138 | 11,7 (4.1) | 46 | 8.6 (4.8) | -4.23 | 182 | 0.001 | 0.72 |
| ADI-R C | 138 | 4,9 (2.7) | 46 | 1.3 (2.6) | -1.47 | 182 | 0.142 | 1.35 |
|  |  |  |  |  |  |  |  |  |
| **Minimally verbal children ADOS Module 2)** | | | | | | | | |
| Age | 227 | 6.74 (3.6) | 339 | 6.12 (3.2) | -2.14 | 564 | .033 | .18 |
| IQ | 112 | 74.67 (26.1) | 134 | 72.18 (35.6) | -.614 | 244 | .540 | .08 |
| IQ- Level | 145 | 4.14 (1.0) | 225 | 3.80 (0.9) | -3.18 | 368 | .002 | .34 |
| ADOS SA | 219 | 10.50 (3.7) | 336 | 3.49 (3.5) | -22.56 | 553 | .000 | 1.95 |
| ADOS RRV | 219 | 2.66 (1.7) | 336 | .67 (0.9) | -17.75 | 553 | .000 | 1.45 |
|  |  |  |  |  |  |  |  |  |
| ADI-R A | 162 | 14.52 (6.44) | 123 | 9.08 (5.58) | -7.51 | 283 | 0.001 | 0.89 |
| ADI-R B | 162 | 12.28 (5.05) | 123 | 6.94 (4.25) | -9.45 | 283 | 0.001 | 1.23 |
| ADI-R C | 162 | 4.92 (2.66) | 123 | 3.01 (2.09) | -6.59 | 283 | 0.001 | 0.77 |
|  |  |  |  |  |  |  |  |  |

IQ-level (According to ICS-10): 1=very superior IQ>129: 2=superior (IQ 115-129), 3=average (IQ 85-114), 4=low (IQ 70-84), 5=mild mental retardation (IQ 50-69), 6=moderate mental retardation (IQ 35-49), 7=severe mental retardation (IQ 20-34). SA=Social Affect, RRV= Restricted Repetitive Behaviours. ADI-R A= Social Interaction, ADI-R B= Communication, ADI-R C= Restricted Repetitive Behaviours. ES= Effect Size (Cohen´s d).

**Table S2.** Psychopathological Description of Participants.

| **Psychopathological description** | | **ASD**  **(n=509)** | **non-ASD**  **(n=523)** |
| --- | --- | --- | --- |
| Cases with comorbid (axis 1 or axis 2) ICD-10 diagnoses | | n=139 (27%) | n=407 (78%) |
|  | F40-F48 Neurotic, stress-related and somatoform disorders | 3 (0.6%) | 5 (1.0%) |
|  | F70-F79 Mental retardation with impairment of behavior | 61 (12.0%) | 56 (10.7%) |
|  | F80-F89 *except* F84 Disorders of psychological development other than pervasive developmental disorders | 115 (22.6%) | 307 (58.6%) |
|  | F90-F98 Behavioral and emotional disorders with onset usually occurring in childhood and adolescence | 55 (10.8%) | 244 (46.6%) |
| Cases with no (axis 1) ICD-10 diagnoses | |  | n=161(31%) |

ASD= autism spectrum disorder; ICD-10= International Statistical Classification of Diseases and Related Health Problems 10th Revision.

**Table S3:** Items and items abbreviations of ADOS and ADI-R.

| **ADOS** | |
| --- | --- |
| ANX | Anxiety |
| ARSC | Amount of Reciprocal Social Communication |
| CONV | Conversation |
| DGES | Descriptive, Conventional, Instrumental, or Informational Gestures |
| ENJ | Shared Enjoyment in Interaction |
| EXPO | Facial Expressions Directed to Others |
| EYE | Unusual Eye Contact |
| GAZE | Integration of Gaze and Other Behaviors During Social Overtures |
| GES | Gestures |
| GIV | Giving |
| IECHO | Immediate Echolalia |
| IJA | Spontaneous Initiation of Joint Attention |
| IMAG | Imagination/Creativity |
| IN | Intonation of Vocalizations or Verbalizations |
| INJ | Self-Injurious Behavior |
| MAN | Hand and Finger and Other Complex Mannerisms |
| NAME | Response to Name |
| NESL | Overall Level of Non-Echoed Spoken Language |
| OACT | Overactivity |
| OQR | Overall Quality of Rapport |
| PLAY | Functional Play With Objects |
| PNT | Pointing |
| QSOV | Quality of Social Overtures |
| QSR | Quality of Social Response |
| REQ | Requesting |
| RINT | Unusually Repetitive Interests or Stereotyped Behaviors |
| RJA | Response to Joint Attention |
| SHO | Showing |
| SINT | Unusual Sensory Interest in Play Material/Person |
| SMILE | Responsive Social Smile |
| SPAB | Speech Abnormalities Associated With Autism |
| STER | Stereotyped/Idiosyncratic Use Of Words or Phrases |
| SVOV | Frequency of Spontaneous Vocalization Directed to Others |
| TAN | Tantrums, Aggression, Negative or Disruptive Behavior |
| UANB | Use of Another´s Body |
| **ADI-R** | |
| ADI-R: A31 | Use of Other´s Body to communicate |
| ADI-R: A49 | Imaginative Play with Peers |
| ADI-R: A50 | Direct Gaze |
| ADI-R: A51 | Social Smiling |
| ADI-R: A52 | Showing and Directing Attention |
| ADI-R: A53 | Offering to Share |
| ADI-R: A54 | Seeking to share Enjoyment With Others |
| ADI-R: A55 | Offering Comfort |
| ADI-R: A56 | Quality of Social Overtures |
| ADI-R: A57 | Range of Facial Expressions Used to Communicate |
| ADI-R: A58 | Inappropriate Facial Expressions |
| ADI-R: A59 | Appropriateness of Social Response |
| ADI-R: A62 | Interest in children |
| ADI-R: A63 | Response to approaches of Other children |
| ADI-R: A64 | Group Play with Peers |
| ADI-R: A65 | Friendships |
| ADI-R: B33 | Stereotyped Utterances and Delayed Echolalia |
| ADI-R: B34 | Social Verbalization/Chat |
| ADI-R: B35 | Reciprocal Conversation |
| ADI-R: B36 | Inappropriate Questions of statements |
| ADI-R: B37 | Pronominal Reversal |
| ADI-R: B38 | Neologism/Idiosyncratic Language |
| ADI-R: B42 | Pointing to Express Interest |
| ADI-R: B43 | Nodding |
| ADI-R: B44 | Head Shaking |
| ADI-R: B45 | Conventional/Instrumental Gestures |
| ADI-R: B47 | Spontaneous Imitation of Actions |
| ADI-R: B48 | Imaginative Play |
| ADI-R: B61 | Imitative Social Play |
| ADI-R: C39 | Verbal Rituals |
| ADI-R: C67 | Unusual Preoccupations |
| ADI-R: C68 | Circumscribed Interests |
| ADI-R: C69 | Repetitive Use of Objects or Interest in Parts of Objects |
| ADI-R: C70 | Compulsions/Rituals; |
| ADI-R: C71 | Unusual Sensory Interests |
| ADI-R: C77 | Hand and Finger Mannerisms |
| ADI-R: C78 | Other Complex Mannerisms or Stereotyped Body Movements |
